# Prevalence and distribution of schistosomiasis in human, livestock, and snail populations in northern Senegal: a One Health epidemiological study of a multi-host system

**DOI:** 10.1016/S2542-5196(20)30129-7

**Published:** 2020-08-12

**Authors:** Elsa Léger, Anna Borlase, Cheikh B Fall, Nicolas D Diouf, Samba D Diop, Lucy Yasenev, Stefano Catalano, Cheikh T Thiam, Alassane Ndiaye, Aidan Emery, Alice Morrell, Muriel Rabone, Momar Ndao, Babacar Faye, David Rollinson, James W Rudge, Mariama Sène, Joanne P Webster

**Affiliations:** a Centre for Emerging, Endemic and Exotic Diseases, Department of Pathobiology and Population Sciences, Royal Veterinary College, University of London, Hertfordshire, UK; b London Centre for Neglected Tropical Disease Research, School of Public Health, Imperial College London, London, UK; c NTD Modelling Consortium, Big Data Institute, University of Oxford, Oxford, UK; d Faculté de Médecine, Pharmacie et Odontologie, Université Cheikh Anta Diop, Dakar, Senegal; e Institut Supérieur de Formation Agricole et Rurale, Université de Thiès, Bambey, Senegal; f Unité de Formation et de Recherche des Sciences Agronomiques, d'Aquaculture et de Technologies Alimentaires, Université Gaston Berger, Saint-Louis, Senegal; g Parasites and Vectors Division, Life Sciences Department, Natural History Museum, London, UK; h National Reference Centre for Parasitology, Research Institute of the McGill University Health Centre, Montreal, QC, Canada; i Communicable Diseases Policy Research Group, London School of Hygiene & Tropical Medicine, London, UK; j Faculty of Public Health, Mahidol University, Bangkok, Thailand

## Abstract

**Background:**

Schistosomiasis is a neglected tropical disease of global medical and veterinary importance. As efforts to eliminate schistosomiasis as a public health problem and interrupt transmission gather momentum, the potential zoonotic risk posed by livestock *Schistosoma* species via viable hybridisation in sub-Saharan Africa have been largely overlooked. We aimed to investigate the prevalence, distribution, and multi-host, multiparasite transmission cycle of *Haematobium* group schistosomiasis in Senegal, West Africa.

**Methods:**

In this epidemiological study, we carried out systematic surveys in definitive hosts (humans, cattle, sheep, and goats) and snail intermediate hosts, in 2016–18, in two areas of Northern Senegal: Richard Toll and Lac de Guiers, where transmission is perennial; and Barkedji and Linguère, where transmission is seasonal. The occurrence and distribution of *Schistosoma* species and hybrids were assessed by molecular analyses of parasitological specimens obtained from the different hosts. Children in the study villages aged 5–17 years and enrolled in school were selected from school registers. Adults (aged 18–78 years) were self-selecting volunteers. Livestock from the study villages in both areas were also randomly sampled, as were post-mortem samples from local abattoirs. Additionally, five malacological surveys of snail intermediate hosts were carried out at each site in open water sources used by the communities and their animals.

**Findings:**

In May to August, 2016, we surveyed 375 children and 20 adults from Richard Toll and Lac de Guiers, and 201 children and 107 adults from Barkedji and Linguère; in October, 2017, to January, 2018, we surveyed 386 children and 88 adults from Richard Toll and Lac de Guiers, and 323 children and 85 adults from Barkedji and Linguère. In Richard Toll and Lac de Guiers the prevalence of urogenital schistosomiasis in children was estimated to be 87% (95% CI 80–95) in 2016 and 88% (82–95) in 2017–18. An estimated 63% (in 2016) and 72% (in 2017–18) of infected children were shedding *Schistosoma haematobium–Schistosoma bovis* hybrids. In adults in Richard Toll and Lac de Guiers, the prevalence of urogenital schistosomiasis was estimated to be 79% (52–97) in 2016 and 41% (30–54) in 2017–18, with 88% of infected samples containing *S haematobium–S bovis* hybrids. In Barkedji and Linguère the prevalence of urogenital schistosomiasis in children was estimated to be 30% (23–38) in 2016 and 42% (35–49) in 2017–18, with the proportion of infected children found to be shedding *S haematobium–S bovis* hybrid miracidia much lower than in Richard Toll and Lac de Guiers (11% in 2016 and 9% in 2017–18). In adults in Barkedji and Linguère, the prevalence of urogenital schistosomiasis was estimated to be 26% (17–36) in 2016 and 47% (34–60) in 2017–18, with 10% of infected samples containing *S haematobium–S bovis* hybrids. The prevalence of *S bovis* in the sympatric cattle population of Richard Toll and the Lac de Guiers was 92% (80–99), with *S bovis* also found in sheep (estimated prevalence 14% [5–31]) and goats (15% [5–33]). In Barkedji and Linguère the main schistosome species in livestock was *Schistosoma curassoni*, with an estimated prevalence of 73% (48–93) in sheep, 84% (61–98) in goats and 8% (2–24) in cattle. *S haematobium–S bovis* hybrids were not found in livestock. In Richard Toll and Lac de Guiers 35% of infected *Bulinus* spp snail intermediate hosts were found to be shedding *S haematobium–S bovis* hybrids (68% shedding *S haematobium*; 17% shedding *S bovis*); however, no snails were found to be shedding *S haematobium* hybrids in Barkedji and Linguère (29% shedding *S haematobium*; 71% shedding *S curassoni*).

**Interpretation:**

Our findings suggest that hybrids originate in humans via zoonotic spillover from livestock populations, where schistosomiasis is co-endemic. Introgressive hybridisation, evolving host ranges, and wider ecosystem contexts could affect the transmission dynamics of schistosomiasis and other pathogens, demonstrating the need to consider control measures within a One Health framework.

**Funding:**

Zoonoses and Emerging Livestock Systems programme (UK Biotechnology and Biological Sciences Research Council, UK Department for International Development, UK Economic and Social Research Council, UK Medical Research Council, UK Natural Environment Research Council, and UK Defence Science and Technology Laboratory).

**Research in context**

**Evidence before this study**

Despite many years of mass administration of the anthelmintic praziquantel to school-aged children, the burden of schistosomiasis remains extremely high in many regions across sub-Saharan Africa. In pursuit of “a world free of schistosomiasis”, the WHO roadmap on neglected tropical diseases set out a comprehensive plan for the control of schistosomiasis, its elimination as a public health problem, and interruption of transmission in selected African regions by 2025. In endemic regions of Asia, animal hosts are considered important zoonotic reservoirs for schistosomiasis. By contrast, the zoonotic component of schistosomiasis transmission has received little consideration in sub-Saharan Africa.

We searched PubMed, Science Direct, and the WHO database for combined studies on human and animal schistosomiasis in Africa using the words “animal” AND “human” AND “schistosomiasis” AND “Africa”, published up to May 23, 2019, with no language restrictions applied. Articles were considered relevant if they assessed the relation between schistosomiasis in humans and naturally infected animals. In sub-Saharan Africa, early studies incorporating livestock data considered the disease in humans and animals as two different systems, and used equivocal morphological traits to distinguish between the various *Schistosoma* species. Studies on the outbreak of schistosomiasis in Corsica, France, have suggested that one of the causative species was a hybrid between *Schistosoma haematobium* and *Schistosoma bovis*, originating in Senegal; however, no local zoonotic reservoir was identified. Other studies of the potential role of animals as zoonotic reservoirs of schistosomiasis in Africa had small sample sizes (<600 humans or animals) with opportunistic sampling in schools for humans and abattoirs for livestock. None of these studies aimed to quantify the disease burden in both human and livestock populations or evaluate of the connection between the two.

**Added value of this study**

This study was performed across 3 years in northern Senegal, using randomised sampling protocols for both human and animal hosts. The prevalence of schistosomiasis was extremely high in both human (up to 88% for urogenital schistosomiasis in children) and livestock populations (up to 94% for *S bovis* in cattle). Viable hybrids between *S haematobium* and *S bovis* occurred frequently, with up to 72% of infected children found to be shedding hybrids. The same hybrids were also found to be shed by snail intermediate hosts, but were not found in sympatric livestock.

To our knowledge, this is the largest and most comprehensive study combining parasitological, epidemiological, and molecular data to evaluate the occurrence and distribution of *Schistosoma* species and hybrids across several potential definitive hosts and snail intermediate hosts in sub-Saharan Africa. Although previously largely ignored, the high prevalence in livestock populations within sub-Saharan Africa could have considerable socioeconomic and welfare consequences for livestock-keeping communities, and also represents a continued risk to human health via zoonotic transmission and hybridisation between livestock and human schistosomes.

**Implications of all the available evidence**

This study highlights the importance of recognising the multi-host, multi-parasite aspects of disease systems under evolutionary pressure. Understanding the interactions within and between *Schistosoma* species and their different hosts in the context of intense anthropogenic environmental change is of crucial importance and should inform public health and animal health measures at local, national, and international levels, to achieve the WHO targets for interrupting transmission of schistosomiasis in sub-Saharan Africa.

## Introduction

Parasitic diseases are a major cause of morbidity and mortality worldwide, and disproportionately affect the poorest populations in sub-Saharan Africa. Many parasites are zoonotic (transmitted from animals to humans), with transmission cycles involving a range of reservoirs that can include the livestock species on which rural livelihoods depend.^1^, ^2^ Guided by the Millennium Development Goals and the Sustainable Development Goals, much progress has been made in reducing the burden of human infection.^3^, ^4^ However, new challenges are emerging as a result of changing environments and populations. This is particularly true for schistosomiasis—a waterborne neglected tropical disease caused by dioecious parasitic trematodes of the *Schistosoma* genus—which is indirectly transmitted to mammalian definitive hosts via freshwater molluscan intermediate hosts. Schistosomiasis has the second highest socioeconomic impact of any parasitic disease (after malaria), and more than 220 million people are currently estimated to be infected, predominantly in low-income and middle-income countries.^5^, ^6^ In pursuit of “a world free of schistosomiasis”, the current WHO roadmap sets goals to control morbidity by 2020, eliminate schistosomiasis as a public health problem by 2025, and to interrupt transmission in member states and selected African countries, by 2025.^7^ Such ambitious goals require an in-depth understanding of the disease context in sub-Saharan Africa and the contribution of all populations to ongoing transmission in endemic zones.

In Asia, efforts to eliminate *Schistosoma japonicum* have recognised animal reservoirs as major drivers of ongoing transmission.^8^ By contrast, in sub-Saharan Africa, *Schistosoma mansoni*, the main cause of intestinal schistosomiasis in humans, is reported to occur only occasionally in non-human primates and rodents.^9^, ^10^, ^11^, ^12^ Furthermore, *Schistosoma haematobium* is believed to be capable of naturally infecting humans only (and very rarely non-human primates^10^), and was traditionally considered to be the sole cause of human urogenital schistosomiasis. In this context, current control programmes in sub-Saharan Africa, which are based on preventive chemotherapy or mass administration of the anthelmintic praziquantel, target only humans (primarily school-aged children) and ignore the potential role of zoonotic reservoirs and the obstacle that they might pose to achievement of control and elimination goals.^13^, ^14^

However, schistosomiasis also affects domestic livestock in sub-Saharan Africa, often in the underprivileged communities most affected by human schistosomiasis.^15^ Intestinal schistosomiasis caused by *Schistosoma bovis, Schistosoma curassoni*, or *Schistosoma mattheei* can lead to enteritis, anaemia, emaciation, and potentially death in cattle, sheep, and goats.^16^ These intestinal livestock schistosome species and the human urogenital species *S haematobium* are members of the *Haematobium* group and frequently overlap in their geographical and host ranges.^10^ Naturally occurring viable hybridisation and introgression (genetic flow from one species to another via repeated backcrossing) between and within human and animal schistosomes—particularly within the *Haematobium* group—are emerging as topics of major importance for global health and disease control.^17^, ^18^, ^19^, ^20^, ^21^, ^22^, ^23^, ^24^, ^25^, ^26^, ^27^ Anthropogenic changes, such as dam construction, migration of people and their animals, and altered agricultural practices, are predicted to increase opportunities for interspecific exposure and co-infection, and therefore hybridisation.^20^, ^22^ A recent outbreak of human schistosomiasis in Corsica, France, was found to be caused by both *S haematobium* and *S haematobium– S bovis* hybrids (closely related to those found in Senegal); however, a local animal reservoir was not identified despite extensive sampling.^17^, ^19^, ^28^ Within sub-Saharan Africa, the potential for livestock hosts to act as a reservoir for human schistosomiasis has not been fully explored.

National statistics in Senegal suggest that the prevalence of urogenital schistosomiasis (the most widespread form of the disease) ranges from 10% in the central regions, where transmission is seasonal, to more than 95% in the Senegal River basin, where transmission is perennial.^29^ By contrast, livestock schistosomiasis has received little attention in Senegal, with no recent, publicly available surveillance data.^25^, ^26^ The primary objective of this study was to elucidate the multi-host, multi-parasite transmission cycle of zoonotic schistosomiasis in sub-Saharan Africa, and the implications for human and animal health, by conducting systematic surveys across multiple potential definitive hosts (humans, cattle, sheep, and goats) and snail intermediate hosts in two putative hybrid hotspot areas of contrasting anthropogenic change in northern Senegal. This incorporated testing the hypothesis that *S haematobium* or *S haematobium* hybrids might be able to infect livestock species, and that (if present) they may be located in, and transmitted via, the urinary system—an aspect of transmission that has not previously been fully explored. Combining parasitological, epidemiological, and molecular approaches within a One Health framework, we evaluated the occurrence and distribution of Schistosoma species and hybrids across potential hosts.

## Methods

### Study sites and populations

**Figure 1:**
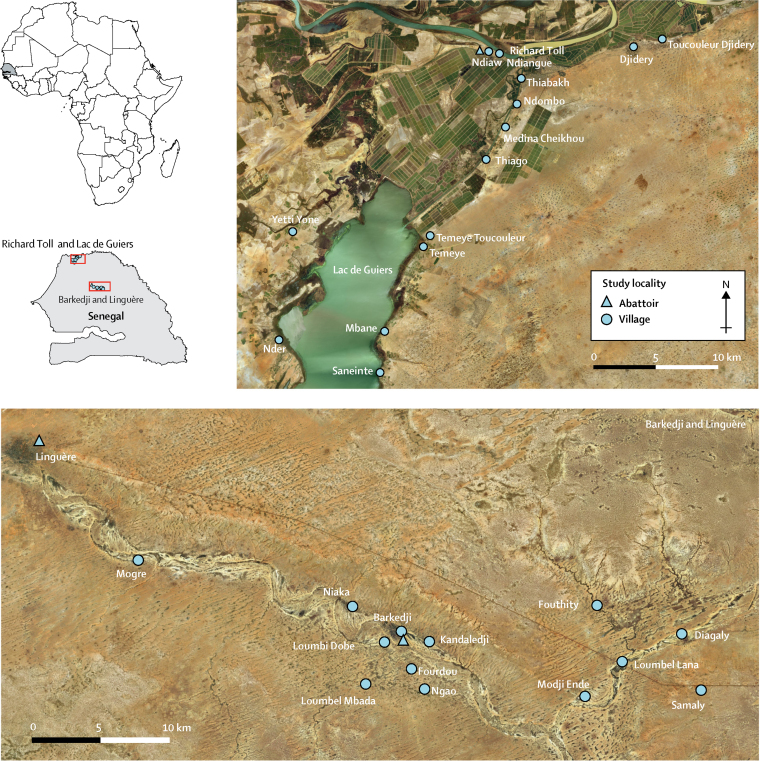
Map of the two study sites in Senegal Study villages and communities are indicated with a circle. Abattoirs are indicated with a triangle.

Epidemiological and parasitological surveys were done in two areas of northern Senegal: Richard Toll and Lac de Guiers in the Senegal River basin, and Barkedji and Linguère in the Vallée du Ferlo (figure 1). Because of the proximity of the Diama Dam, the Richard Toll and Lac de Guiers area has undergone substantial land-use changes, with permanent alterations to the local ecology. Desalination and creation of irrigation canals has facilitated expansion of habitats for snail intermediate hosts and increased sharing of water contact points by communities and their animals. In this area, permanent water bodies and persistent water contact maintains transmission of the disease throughout the year. By contrast, in Barkedji and Linguère, temporary ponds are an important water source for human populations and their animals. These water sources disappear completely during the dry season, interrupting transmission of schistosomiasis and necessitating seasonal migration by a large proportion of livestock-keeping communities.

Following the construction of the Diama Dam in 1988, a well documented outbreak of human *S mansoni* intestinal schistosomiasis occurred in Richard Toll and Lac de Guiers, and both *S mansoni* and *S haematobium* are now endemic across the region.^30^, ^31^ Furthermore, zoonotic hybrids between *S bovis* and *S haematobium* have been observed in the human populations of both the Richard Toll and Lac de Guiers area and the Barkedji and Linguère area.^18^, ^25^, ^30^ On the basis of these previous studies, local knowledge, and preliminary evaluation of water access points, 14 villages or communities in the Richard Toll and Lac de Guiers area and 13 villages in Barkedji and Linguère were selected for sampling of humans, livestock (cattle, sheep, and goats), and snails (figure 1).

All children in the study villages aged 5–17 years and enrolled in school were eligible to be selected, with samples selected randomly using random selection functions from Excel spreadsheets of school registers, and the sample size for each school proportional to the number of children in the school. Adults (aged 18–78 years) were self-selecting volunteers recruited in households within each village.

Our surveys (May to August, 2016, and October, 2017, to January, 2018) were timed such that they were not closely preceded by mass drug administration activities carried out by the Senegalese national schistosomiasis control programme (which took place in Richard Toll and Lac de Guiers in December, 2015, and in December, 2016, to January, 2017, and in Barkedji and Linguère in December, 2015).

All animals (cattle, sheep, and goats) routinely slaughtered as part of the normal work of the abattoirs and available for inspection at the time of the surveys were examined post mortem. In the abattoir survey we obtained *Schistosoma* adult worms from cattle, sheep, and goat specimens (which are not accessible ante-mortem), to identify the location and species of schistosome eggs in livestock hosts, and to examine the potential for urogenital schistosomiasis and transmission via the urinary route in these species. Because of the wide geographical area from which animals might be brought to the abattoir, and the potential biases in both the age and health profile of the animals presented, abattoir data were not considered sufficiently representative of livestock populations sympatric to our study villages to enable accurate prevalence estimates. However, abattoir data were used to inform estimates of the performance of diagnostic tests for the survey of living livestock.

Sampling of living livestock enabled a larger and more representative sample (than the abattoir survey) of sympatric livestock known to belong to, and share water contact points with, our study communities, giving a more accurate picture of the prevalence of schistosomiasis in livestock and which schistosome species are co-circulating. A preliminary study was conducted in Richard Toll and Lac de Guiers in which diagnostic test methods were refined and a census of livestock-owning households was undertaken to estimate livestock populations in all villages. A second survey was then carried out in Richard Toll and Lac de Guiers to assess the prevalence of schistosomiasis. Sample sizes for each species per village were weighted according to the estimated population size of that species in the village. Initial randomisation was carried out at the unit level (owner in this case), with a maximum of five animals of each species then randomly sampled from each selected owner (fewer if the owner had fewer than five animals). Randomisation was carried out using random number generators.

In Barkedji and Linguère, the sample size per village was weighted according to the number of households, with the same number of animals sampled for each livestock owner in the village who was willing to participate in the study. A reluctance within Barkedji and Linguère communities to disclose the exact number of animals owned by a household prevented a livestock census and local government veterinary records were used for population estimates. Additionally, two moribund sheep slaughtered during the live animal survey in Barkedji and Linguère were examined at post mortem to quantify worm burden.

Five malacological surveys of snail intermediate hosts were carried out at each site in open water sources used by the communities and their animals.

Ethical approval was provided by Imperial College London (London, UK; application 03.36), the Clinical Research Ethical Review Board (CRERB) at the Royal Veterinary College (London, UK; application URN20151327), and the Comité National d'Ethique pour la Recherche en Santé (Dakar, Senegal; application SEN15/68). Written informed consent was obtained from all adult participants, children's parents or guardian(s), and livestock owners. Infected adults and children were treated with 40 mg/kg praziquantel. Livestock owners were offered standard anthelmintics for infected animals.

### Procedures

One urine and one stool sample were collected in the morning from each human participant. For diagnosis of urogenital schistosomiasis, two 10 mL urine filtrations were performed on each sample and examined microscopically to detect the presence of, and quantify, schistosome eggs. Filters that were positive for schistosome eggs were placed in fresh water and exposed to light to facilitate egg hatching into miracidia. For intestinal schistosomiasis, two Kato-Katz slides were prepared from each faecal sample. Kato-Katz-positive stool samples were processed using an adapted miracidia hatching technique (MHT; appendix p 1). Free-swimming miracidia from positive urine and stool samples were individually pipetted onto Whatman Indicating FTA Classic Cards (GE Healthcare Life Sciences, Buckinghamshire, UK) for DNA storage and molecular analysis.

In the abattoir samples, mesenteric and rectal blood vessels were visually inspected for the presence of adult worms at post-mortem, and sections of liver, spleen, lungs, and kidney were collected and processed following an adapted MHT (appendix p 1). Rectal faeces were obtained; two Kato-Katz slides were examined from each sample and MHT was performed on all samples. Bladders and associated vasculature were examined visually for the presence of worms, and urine obtained for filtration. If no urine was present, sections of bladder were taken and processed in the same way as the organ samples. All adult worms (single males, females, and split couples) were stored in RNA*later* (Sigma-Aldrich, UK). Following urine filtration and MHT of faeces and tissue samples, free-swimming miracidia were individually pipetted onto Whatman Indicating FTA Classic Cards for storage and analysis.

Rectal faecal sampling was performed for all animals included in livestock surveys, and free-catch urine samples were collected wherever possible. Samples were processed and miracidia collection performed in the same manner as for the human samples. Additionally, one-third of faecal samples were randomly selected and processed following Kato-Katz and MHT protocols in parallel.

Malacological surveys of snail intermediate hosts were carried out following standardised snail scooping protocols.^32^ All *Bulinus* spp and *Biomphalaria* spp snails were individually exposed to light in fresh water to induce *Schistosoma* spp cercariae shedding. Free-swimming cercariae shed by infected snails were individually pipetted onto Whatman Indicating FTA Classic Cards for storage and analysis.

DNA was extracted from individual adult worms stored in RNA*later*, and from individual miracidia and cercariae stored on Whatman Indicating FTA Classic Cards.^33^ Individual *Schistosoma* DNA extracts were characterised by amplification of a partial fragment of the mitochondrial cytochrome c oxidase subunit 1 (*cox1*) and the complete nuclear ribosomal DNA internal transcribed spacer (ITS; appendix p 1). PCR fragments were sequenced by Eurofins Genomics (Cologne, Germany) using original primers. Sequences were manually edited and assembled using CodonCode Aligner, version 7.0.1, and compared with *Schistosoma* reference sequences to confirm species.^9^, ^18^, ^25^ Molecular sequences from representative samples from the different species were deposited in GenBank (accession numbers MT580946-963, MT579420-449 and MN593376, MN593380, MN593384, MN593388, MN593392, MN593396, MN593400, and MN593404; MN593376 to MN593404 were deposited as part of a previous study^11^).

Hybrid 1 (where the *S bovis cox1* mitochondrial DNA profile is associated with the *S haematobium* nuclear ITS profile) represents miracidia that are the product of repeated backcrossing of hybrids with *S haematobium*, resulting in biased homogenisation towards this species and ITS sequences that appear as just one species. By contrast, miracidia designated as hybrid 2 exhibit either *S bovis* or *S haematobium cox1* profiles associated with both *S haematobium* and *S bovis* parental nuclear ITS copies, appearing as double peaks on the four species-specific mutation sites on chromatograms. The peak height representing each species can vary, with some hybrids having a higher peak for one species and some hybrids having equal peak heights for both species. Hybrid 2 therefore encompasses first generation (F1) hybrid miracidia arising from the cross-species pairing of *S haematobium* with *S bovis*, and recent backcrossed hybrids (appendix p 2).

### Statistical analysis

Sample sizes for humans were calculated based on the ability to detect a significant minimum difference in egg reduction rate between children with zoonotic *S haematobium*–*S bovis* hybrid infections (pre-praziquantel) from those with single *S haematobium* infections (controlling for locality and infection intensities). Based on the recent literature,^34^, ^35^ we predicted to achieve a 90% egg reduction rate in single *S haematobium* infections and 70% for hybrid infections. Based on this we required a sample size of approximately 180 infected children and adults to detect such a difference in egg reduction rate.

Sample sizes for the animal surveys, based on a cluster survey design, were determined on the basis that we aimed to obtain a set precision on our livestock prevalence estimate, but also to evaluate, with a set degree of certainty, whether novel zoonotic hybrids occur in livestock at all. We originally estimated a prevalence of 20% for zoonotic hybrids; in order to be 95% certain this is within 10% of the population value, we aimed to achieve a sample size of 200 cattle, 200 sheep, and 200 goats per region and per survey.

In humans, the prevalence of urogenital and intestinal schistosomiasis was estimated by egg detection in urine filters and Kato-Katz slides, respectively. Estimates were adjusted using a Bayesian framework to allow for the uncertainty around the sensitivity of a single diagnostic test (appendix p 2), with informative priors taken from the literature (appendix p 5). The same framework was used to estimate prevalence in snails (appendix pp 2, 5). The diagnostic sensitivity of Kato-Katz and MHT tests in livestock was estimated using abattoir data, and then used in Bayesian estimation of true prevalence among livestock from the live animal survey data (appendix pp 2–5). All statistical tests and Bayesian analyses were done in R version 3.5.1. Bayesian simulations were run with JAGS version 4.3.0 using Markov Chain Monte Carlo simulations (two chains, 200 000 iterations, burn-in of 5000, and thinning interval of 40) implemented in R version 3.5.1 using the rjags and coda packages.^36^, ^37^, ^38^ Differences between the proportion of infected hosts were analysed using Pearson's χ^2^ test or Fisher's exact test. Potential correlation between the village-level density of infected animals and the proportion of hybrids in humans was assessed using Spearman's correlation coefficient. 95% CIs presented for the observed proportions of schistosome genotypes were calculated using the Clopper-Pearson exact method. Statistical tests were considered significant when p≤0·05.

### Role of the funding source

The funders of the study had no role in study design, data collection, data analysis, data interpretation, or writing of the report. The corresponding author had full access to all the data in the study and had final responsibility for the decision to submit for publication.

## Results

We carried out two human surveys at each site. The first included 375 children and 20 adults from the Richard Toll and Lac de Guiers area (surveyed from May 6 to June 10, 2016), and 201 children and 107 adults from Barkedji and Linguère (surveyed on Aug 2–25, 2016). The second survey included 386 children and 88 adults from Richard Toll and Lac de Guiers (surveyed between Dec 6, 2017, and Jan 29, 2018), and 323 children and 85 adults from Barkedji and Linguère (surveyed in Oct 17–30, 2017).

**Figure 2:**
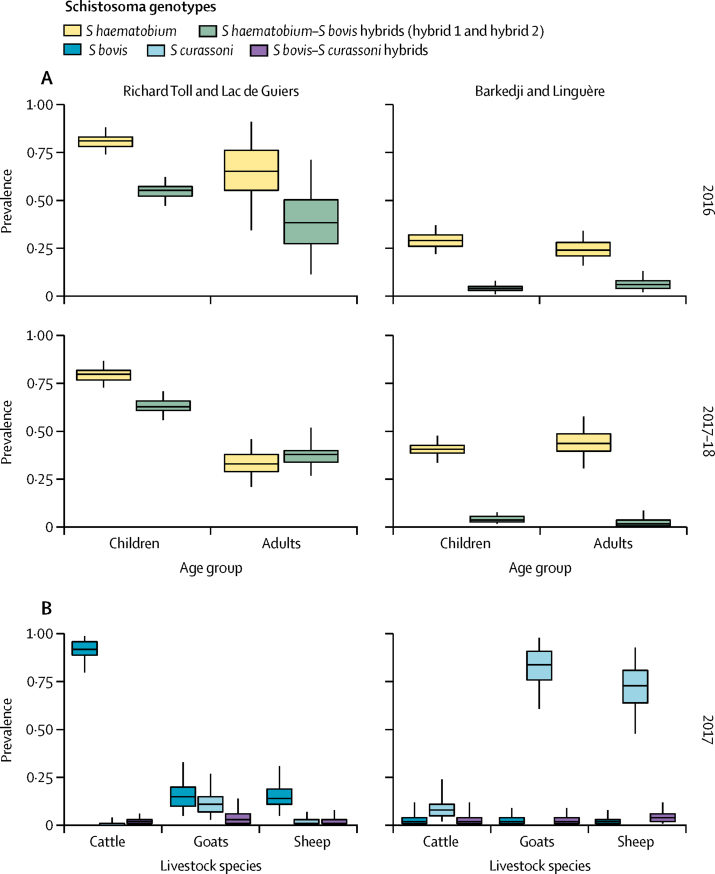
Estimated prevalence of schistosome genotypes Estimated prevalence of schistosome genotypes in human (A) and livestock (B) populations of the Richard Toll and Lac de Guiers area and the Barkedji and Linguère area. Box plots represent median, IQR, and range. Data are tabulated in the appendix (pp 6–7).

Across all villages in both sites and surveys, in adults and children, 2575 miracidia collected from 472 egg-positive urine samples were successfully analysed (table 1). The *S haematobium* profile was observed for 1969 (76%) of the genotyped miracidia. 606 (24%) miracidia displayed both *S haematobium* and *S bovis* or both *S bovis* and *S curassoni* signals and were designated as hybrids. Two different *S haematobium–S bovis* hybrid profiles were identified and excretion of multiple genotypes by an individual was commonly observed. The most frequent hybrid profile (554 [91%] of 606 genotyped hybrid miracidia) was that designated as hybrid 1. Molecular data were combined with overall prevalence to give estimates by genotype (figure 2A, appendix pp 6–7).

**Table 1:** Human population survey results

	**Urinogenital schistosomiasis**					**Intestinal schistosomiasis**	
	Urine filtration positive (n/N)	Median posterior prevalence (BCI)	Proportion of infected individuals with *Schistosoma haematobium* miracidia (n*)	Proportion of infected individuals with hybrid miracidia (n*)	Intensity of infection (median number of eggs per 10 mL [IQR; range])	Kato-Katz positive (n/N)	Median posterior prevalence (BCI)
**Richard Toll and Lac de Guiers, 2016**							
Children	264/375	0·87 (0·80–0·94)	0·94 (168)	0·63 (168)	28 (6–90; 1–990)	45/318	0·20 (0·14–0·32)
Adults	13/20	0·79 (0·52–0·97)	1 (4)	0·5 (4)	5 (3–8; 1–58)	0/20	0·05 (0·00–0·24)
**Richard Toll and Lac de Guiers, 2017–18**							
Children	275/386	0·88 (0·82–0·95)	0·91 (166)	0·72 (166)	10·5 (2–41; 1–433)	35/292	0·17 (0·11–0·28)
Adults	29/88	0·41 (0·30–0·54)	0·85 (14)	1 (14)	2 (1–26; 1–48)	0/41	0·02 (0·00–0·13)
**Barkedji and Linguère, 2016**							
Children	48/201	0·30 (0·23–0·38)	1 (36)	0·11 (36)	17 (6–65; 1–500)	0/203	0·00 (0·00–0·03)
Adults	22/107	0·26 (0·17–0·36)	1 (14)	0·21 (14)	3 (1–20, 1–295)	0/111	0·01 (0·00–0·05)
**Barkedji and Linguère, 2017–18**							
Children	109/323	0·42 (0·35–0·49)	1 (54)	0·09 (54)	30 (2–113; 1–500)	0/289	0·00 (0·00–0·02)
Adults	32/85	0·47 (0·34–0·60)	1 (16)	0 (16)	6 (3–20; 1–200)	0/58	0·02 (0·00–0·09)

Summary of human population surveys in Richard Toll and Lac de Guiers, and Barkedji and Linguère, in 2016 and 2017–18, including Bayesian estimation of prevalence of urinary and intestinal schistosomiasis. Median *Schistosoma* eggs per mL of urine is given as an indicator of infection intensity. Children were aged 5–17 years and adults aged 18–78 years. BCI=Bayesian credible interval.

* n represents the number of positive individuals for whom molecular material was analysed.

The proportion of children who tested positive for urogenital schistosomiasis was significantly higher in Richard Toll and Lac de Guiers than in Barkedji and Linguère for both surveys (264 [70%] of 375 *vs* 48 [24%] of 201, 1 df, χ^2^=112·2, p<0·0001, in 2016; 275 [71%] of 386 *vs* 109 [34%] of 323, 1 df, χ^2^=98·1, p<0·0001, in 2017–18; table 1). No significant difference in the proportion of children who tested positive for urogenital schistosomiasis in Richard Toll and Lac de Guiers was found between the two surveys (1 df, χ^2^=0·03, p=0·86; table 1); however, the proportion of infected children was significantly higher in the 2017–18 survey than in the 2016 survey in Barkedji and Linguère (1 df, χ^2^=5·3, p=0·02).

Across both surveys, 227 (68%; 95% CI 63–73) of 334 samples from infected children in Richard Toll and Lac de Guiers had hybrid miracidia, compared with 9 (10%; 5–18) of 90 infected children in Barkedji and Linguère. The proportion of infected children shedding hybrid eggs or miracidia was significantly higher in Richard Toll and Lac de Guiers than in Barkedji and Linguère (0·63 *vs* 0·11 in 2016 and 0·72 *vs* 0·09 in 2017–18; Fisher's exact test p<0·0001 for both surveys). Molecular material was available from fewer adults than children (18 in Richard Toll and Lac de Guiers and 30 in Barkedji and Linguère); however, a higher proportion of adults were also shedding hybrids in Richard Toll and Lac de Guiers (16 [89%; 95% CI 65–99]) than in Barkedji and Linguère (3 [10%; 2–27]; table 1).

For both surveys, 546 (31%; SD 31; range 0–100) of 1764 miracidia from children who tested positive in Richard Toll and Lac de Guiers, and 11 (2%; SD 7·4; range 0–50) of 548 miracidia from children who tested positive in Barkedji and Linguère were typed as hybrids. In adults, 42 (42%; SD 25; range 0–67) of 99 miracidia in Richard Toll and Lac de Guiers and 7 (4%; SD 18; range 0–83) of 164 miracidia in Barkedji and Linguère were typed as hybrids.

23 miracidia were analysed from four positive faecal samples from children in Richard Toll and Lac de Guiers. Only *S mansoni* was identified, and no *S haematobium, S bovis*, or hybrids were found in human faeces (table 1). No adults in Richard Toll and Lac de Guiers and no children or adults in Barkedji and Linguère were positive for faecal schistosome eggs.

91 cattle, 94 sheep, and 193 goats were examined post-mortem in abattoir surveys carried out across the two study areas between November, 2015, and April, 2018 (table 2). 1831 worms and 746 miracidia from 115 infected animals were successfully analysed. Adult schistosome worms were found in the mesenteric blood vessels, and miracidia were hatched from liver, lungs, and faecal samples. *S bovis* adult worms were found within the vesical blood vessels in one cow; however, no eggs or miracidia were isolated from the urine of this animal, nor from any other urine or bladder samples.

**Table 2:** Abattoir survey results

	Number post-mortem positive, n/N*tested (proportion)	**Proportion of positive animals infected with *Schistosoma bovis* (n**†**)**	**Proportion of positive animals infected with *Schistosoma curassoni* (n**†**)**	**Proportion of positive animals infected with *S bovis–S curassoni* hybrids (**n†**)**
**Richard Toll and Lac de Guiers**				
Cattle	49/60 (0·82)	0·94 (46)	0·17 (46)	0·09 (46)
Goats	13/103 (0·13)	0·90 (10)	0 (10)	0·10 (10)
Sheep	14/69 (0·20)	0·46 (11)	0·73 (11)	0·18 (11)
**Barkedji and Linguère**				
Cattle	25/31 (0·81)	0·22 (23)	0·87 (23)	0·57 (23)
Goats	22/90 (0·24)	0 (17)	1 (17)	0 (17)
Sheep	8/25 (0·32)	0 (8)	0·88 (8)	0·13 (8)

Number of post-mortem specimens positive for schistosomiasis and *Schistosoma* genotypes identified are indicated.

* Number of animals positive for schistosome adult worms, eggs, or miracidia in organs, faeces, or both.

† Number of infected animals with molecular material successfully analysed.

The median number of worms isolated per host during the abattoir survey was 23 (range 1–714) in cattle, 7 (1–315) in goats, and 2 (1–11) in sheep. Additionally, more than 300 worms were counted in the two sheep slaughtered in Barkedji and Linguère during the live animal survey. We identified *S bovis, S curassoni*, and *S bovis*–*S curassoni* hybrids in cattle, sheep, and goats in both sites. No *S haematobium* or *S haematobium* hybrid worms or miracidia were isolated from abattoir specimens. Most animals in Richard Toll and Lac de Guiers were infected with *S bovis* (57 [85%] of 67 infected animals), whereas most animals in Barkedji and Linguère were infected with *S curassoni* (44 [92%] of 48 infected animals). Estimated diagnostic test sensitivities (Kato-Katz and MHT) in livestock species based on abattoir data are given in the appendix (p 3).

Urine samples from 69 cattle, 27 goats, and 252 sheep from both areas were tested. No urine samples from any animal species were positive for schistosome eggs or miracidia (table 3).

**Table 3:** Livestock population survey results

	**Kato-Katz positive (n/N)**	MHT positive (n/N*)	**Median posterior prevalence of schistosomiasis**†**(95% BCI)**	**Covariance of disease-positive animals (95% BCI)**	**Proportion of infected animals shedding *S bovis* (n**‡**)**	**Proportion of infected animals shedding S *curassoni* (n**‡**)**	**Proportion of infected animals shedding *S bovis–S curassoni* hybrids (n**‡**)**
**Richard Toll and Lac de Guiers, 2017**							
Cattle	62/203	57/70	0·94 (0·81–1·00)	0·04 (0·00–0·07)	1 (85)	0 (85)	0·01 (85)
Goat	12/189	5/64	0·26 (0·13–0·48)	0·03 (0·00–0·10)	0·6 (5)	0·40 (5)	0 (5)
Sheep	5/196	4/68	0·16 (0·07–0·34)	0·03 (0·00–0·11)	1 (7)	0 (7)	0 (7)
**Barkedji and Linguère, 2016**							
Cattle	9/87	2/32	0·29 (0·12–0·59)	0·03 (0·00–0·12)	0 (2)	1 (2)	0 (2)
Goat	7/152	2/53	0·17 (0·06–0·37)	0·04 (0·00–0·13)	0 (1)	1 (1)	0 (1)
Sheep	12/146	3/44	0·34 (0·16–0·64)	0·02 (0·00–0·08)	0 (2)	1 (2)	0·50 (2)
**Barkedji and Linguère, 2017**							
Cattle	4/192	2/70	0·09 (0·03–0·23)	0·04 (0·00–0·15)	NA	NA	NA
Goat	42/205	29/72	0·86 (0·62–0·99)	0·13 (0·08–0·16)	0 (30)	1 (30)	0 (30)
Sheep	26/204	24/68	0·77 (0·52–0·98)	0·08 (0·03–0·12)	0 (33)	0·97 (33)	0·03 (33)

Because of the poor performance of the Kato-Katz test alone in all livestock species, prevalence estimates are based on animals with MHT and Kato-Katz tests carried out in parallel. MHT=miracidia hatching test. BCI=Bayesian credible interval.

* Random subset of animals with MHT and Kato-Katz tests performed in parallel.

† Based on Kato-Katz and MHT results from random subset of animals with both tests carried out in parallel.

‡ Number of positive animals with miracidia analysed.

After a preliminary livestock survey in Richard Toll and Lac de Guiers between Nov 13, 2015, and Jan 22, 2016, to refine diagnostic test methods and conduct a census of livestock-owning households, a second survey was carried out between Feb 26, 2017, and May 29, 2017. We analysed 599 miracidia from 114 MHT-positive faecal samples obtained from cattle, goats, and sheep from Richard Toll and Lac de Guiers. The proportions of each livestock species found to be shedding *S bovis, S curassoni*, and *S bovis*–*S curassoni* hybrids were combined with the Bayesian prevalence estimates to give the prevalence estimate by genotype (figure 2B). A high prevalence of schistosomiasis was identified in cattle, with a median posterior prevalence estimate of 94% (95% Bayesian credible interval [BCI] 81–100; table 3). All positive cattle from Richard Toll and Lac de Guiers with molecular material analysed were shedding *S bovis*.

In Barkedji and Linguère, 87 cattle, 152 goats, and 146 sheep were sampled between April 4 and April 7, 2016, and 192 cattle, 205 goats, and 204 sheep were sampled between Jan 17 and Feb 21, 2017. We analysed 314 miracidia from 68 positive animals in Barkedji and Linguère across the two surveys. Most (67 [99%] of 68) schistosome infections in the livestock of Barkedji and Linguère were *S curassoni* (figure 2B).

No correlation was found at the village level between the estimated density of infected livestock and the proportion of human samples with *S haematobium*–*S bovis* hybrids (Spearman p=0·52) or estimated prevalence of hybrids in the human population (Spearman p=0·80).

Across five malacological surveys undertaken between November, 2015, and April, 2018, 2532 *Bulinus truncatus* and *Bulinus globosus* snails were collected from Richard Toll and Lac de Guiers. 88 snails were infected with *Schistosoma* spp, representing an adjusted pooled prevalence estimate of 3·71% (95% BCI 2·98–4·55; table 4). From infected *Bulinus* snails, 511 individual cercariae were analysed; snails were shedding *S haematobium, S bovis*, and *S haematobium*–*S bovis* hybrids, with 15 snails shedding cercariae genotypes from two or more species, suggesting co-infection. Cercariae profiles from infected *Bulinus* snails in Richard Toll and Lac de Guiers were similar to those of miracidia shed by humans in this region, with both hybrid 1 and hybrid 2 *S haematobium*–*S bovis* hybrids identified. Additionally, 407 *Biomphalaria pfeifferi* snails were collected, of which nine were shedding *S mansoni* cercariae (estimated prevalence 2·53%, 95% BCI 1·25–4·49).

**Table 4:** Snail survey results

	**Number of snails**	**Schistosoma haematobium**	**Schistosoma bovis**	***S haematobium–S bovis* (hybrid 1)**	***S haematobium–S bovis* (hybrid 2)**	**S curassoni**	**S mansoni**	**Total infected snails (n [median Bayesian posterior prevalence estimate, 95% BCI])**
**Richard Toll and Lac de Guiers**								
*Bulinus truncatus* and *Bulinus globosus*	2532	60 (2·37%)	15 (0·59%)	25 (0·98%)	6 (0·24%)	0	0	88 (3·71, 2·98–4·55)
*Biomphalaria pfeifferi*	407	0	0	0	0	0	9 (2·21%)	9 (2·53, 1·25–4·49)
**Barkedji and Linguère**								
*Bulinus umbilicatus*	4694	6 (0·13%)	0	0	0	15 (0·32%)	0	21 (0·49, 0·31–0·72)

Summary of number (%) of *Bulinus* and *Biomphalaria* snails shedding each schistosome genotype over five malacological surveys. BCI=Bayesian credible interval. Hybrid 1 (where the *S bovis cox1* mitochondrial DNA profile is associated with the *S haematobium* nuclear ITS profile) represents miracidia that are the product of repeated backcrossing of hybrids with *S haematobium*, resulting in biased homogenisation towards this species and ITS sequences that appear as just one species. Hybrid 2 miracidia exhibit either *S bovis* or *S haematobium cox1* profiles associated with both *S haematobium* and *S bovi*s parental nuclear ITS copies, appearing as double peaks on the four species-specific mutation sites on chromatograms.

In Barkedji and Linguère, the snail species involved in transmission of schistosomiasis is *Bulinus umbilicatus.* 4694 *B umbilicatus* were collected between November, 2015, and April, 2018; 21 were shedding schistosome cercariae (median prevalence estimate 0·49%, 95% BCI 0·31–0·72; table 4). 149 individual cercariae were analysed, which identified 15 snails shedding *S curassoni* and six shedding *S haematobium.* No *B globosus, B truncates*, or *Biomphalaria* spp were found in Barkedji and Linguère, and no *B umbilicatus* were shedding hybrid cercariae or *S bovis*.

## Discussion

In this study, we show that urogenital schistosomiasis remains a major public health problem in northern Senegal despite school-based interventions being in place since 2006. We identified Richard Toll and Lac de Guiers as a region with a very high prevalence of urogenital schistosomiasis (as high as 88% in children) and *S haematobium*–*S bovis* hybrids (72% of infected children in the 2017–18 survey). However, we observed substantially lower levels of *S mansoni* infections in Richard Toll and Lac de Guiers than in previous studies (2–20% in our study *vs* 79–100% reported in 2013^31^) and no hybrids were observed in human faeces.^18^ Although the prevalence of urogenital schistosomiasis was still fairly high in Barkedji and Linguère (estimated 42% in children in the 2017–18 survey), we found a significantly lower proportion (21%) of infected people shedding hybrids than in Richard Toll and Lac de Guiers.

Our results also show that livestock schistosomiasis represents a major animal health problem in our study regions, with direct and indirect impact on owners' livelihoods that should not be overlooked.^15^ In Richard Toll and Lac de Guiers, the predominant livestock schistosome was *S bovis*, with particularly high prevalence in cattle (estimated 94%). By contrast, *S bovis* was observed in only a few abattoir specimens in Barkedji and Linguère, probably representing animals imported into the area, and no snails or live animals were found to be shedding *S bovis. S curassoni* was the predominant livestock schistosome species in this area, with the highest prevalence in small ruminants (estimated 86% in goats and 77% in sheep in the 2017 survey).

Despite systematic sampling of more than 1500 live cattle, sheep, and goats in addition to thorough post-mortem sampling of 378 animals across both study sites, we found no evidence of livestock species being infected with *S haematobium,* or *S haematobium*–*S bovis* or *S haematobium*–*S curassoni* hybrids. Previous studies have hypothesised that, if *S haematobium* or hybrids were to infect livestock species, they might migrate to the urogenital system, and eggs might be transmitted via the urinary route.^25^, ^39^ Although this migration and adaptation to urinary transmission cannot be ruled out, we did not find any evidence to support this hypothesis following thorough examination of the bladder and associated vasculature of animals post-mortem and urine samples from living and abattoir specimens.

**Figure 3:**
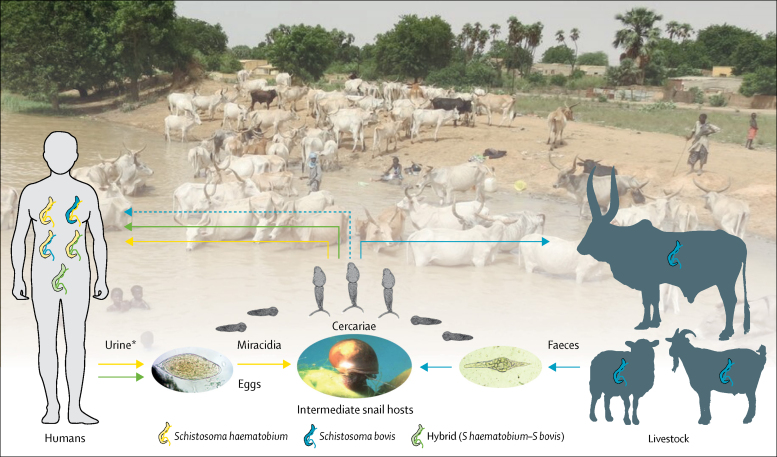
Proposed lifecycle of *Schistosoma haematobium* group in hybrid zones in West Africa—a multi-host, multi-parasite transmission cycle The livestock schistosome *Schistosoma bovis* (blue), is present in cattle, sheep, and goats, and transmission is maintained within these species (egg, miracidia, and cercarial shedding of *S bovis* shown as blue arrows). Evidence indicates that *S bovis* cercariae are able to infect humans (dashed blue line), but unable to complete their lifecycle in the human host unless paired with the human schistosome *S haematobium* (yellow). This cross-species pairing leads to viable hybrid eggs and miracidia (green arrows), which are able to infect snail intermediate hosts and re-infect human hosts. In human hosts, repeated backcrosses and introgression lead to the complex range of miracidia genotypes shed in hybrid zones. *Faecal transmission of *S haematobium–S bovis* hybrids is also possible.

The observed pattern of a high prevalence of *S haematobium*–*S bovis* hybrids in humans in Richard Toll and Lac de Guiers together with a high prevalence of *S bovis* in livestock (compared with lower prevalence of *S haematobium*–*S bovis* in humans and no sympatric transmission of *S bovis* in local livestock in Barkedji and Linguère), identifies a possible association between the presence of *S bovis* in local livestock and the occurrence of *S haematobium*–*S bovis* hybrids in the local human population. Given that *S haematobium*–*S bovis* hybrids have not been observed to be transmitted by any definitive host species other than humans, our results suggest that the initial inter-specific pairing necessary for the formation of F1 hybrids occurs via zoonotic spillover of *S bovis* from a livestock reservoir into human hosts in areas where *S haematobium* and *S bovis* are co-endemic (although a role for rodents and non-human primates cannot be excluded^9^, ^10^, ^28^). Further evidence is provided by our observation of early-generation hybrids (hybrid 2) shed by human hosts, including those with a profile consistent with that expected for F1 hybrids. F1 hybrids accounted for a very low proportion of the hybrid miracidia isolated, suggesting that zoonotic spillover of *S bovis* is either very infrequent or only occasionally leads to patent infections.^26^, ^27^ This observation is consistent with the rare identification of *S bovis* being shed by humans (a single patient identified) in the Corsica outbreak.^17^ However, even if zoonotic transmission from livestock occurs only infrequently, the impact of such rare events can be epidemiologically significant, and the number and impact of these events often increase under evolutionary pressure and as systems near elimination.^40^, ^41^ Indeed, if resistance to praziquantel or similar evolutionarily advantageous traits were to arise in the animal population and be transmitted to the human population via hybridisation, isolated spillover events would become a significant obstacle to elimination efforts. Therefore, in view of our findings, we propose a revised *Haematobium* group transmission cycle, representing the multiple definitive hosts, multiple schistosome species, and the interactions between them (figure 3).

Although co-endemic transmission of *S haematobium* and *S bovis* (followed by co-infection by both species in the same host) is necessary for formation of new hybrids, contrasting ecology is also likely to contribute to differences in the occurrence of hybrids between the two study sites. For example, major anthropogenic changes have occurred recently in Richard Toll and Lac de Guiers, transmission is perennial via permanent water access points in Richard Toll and Lac de Guiers in contrast to the temporary ponds and seasonal transmission in Barkedji and Linguère, and perhaps most importantly, the two areas have differing sympatric snail intermediate host species. Field and laboratory studies have suggested that the intermediate snail host species present in Richard Toll and Lac de Guiers (*B globosus* and *B truncatus*) can be infected by a wide range of schistosome genotypes.^42^, ^43^ The observed range of cercariae genotypes was very similar to those of the miracidia shed by human and livestock populations in Richard Toll and Lac de Guiers, which supports the assumption that the *Bulinus* spp snails present in this region are indeed capable of transmitting the complex array of schistosome genotypes shed by definitive hosts. By contrast, *B umbilicatus* was not observed to shed hybrids in Barkedji and Linguère, suggesting that the few observed *S haematobium*–*S bovis* hybrid cases have probably been imported into the area (which is a region with substantial seasonal movement of both people and animals).

Both *S bovis* and *S curassoni* are able to infect a range of livestock hosts, and the ability of parasites and pathogens to infect multiple host species is a risk factor for disease emergence in humans.^1^ *S bovis*–*S curassoni* hybrids can be zoonotic,^21^ and we observed these hybrids in livestock in both study sites despite their respective transmission zones not overlapping. This observation suggests that both regional and international migration of animals and people might increase opportunities for hybridisation, with potential consequences for disease emergence in the human population. The recent identification of *B pfeifferi* in Lake Malawi (which was not previously observed in this area), and consequent emergence of intestinal schistosomiasis and hybrids (*S haematobium*–*S bovis* and *S haematobium*–*S mattheei*) in the human population, has highlighted the potential for schistosome species to invade new areas in the presence of a suitable snail host.^24^, ^44^ Likewise, with current global changes and the potential for hybridisation to extend intermediate and definitive host ranges, the recent outbreak of schistosomiasis in Corsica has further shown the role of migration in the expanding geographical range of schistosome hybrids.^17^, ^19^, ^28^

The zoonotic component of schistosomiasis in sub-Saharan Africa has been completely overlooked by control programmes, and evidence is building that it might be more significant than previously assumed. Although our study included only two study sites in northern Senegal, our results provide valuable insights into potential drivers for the creation and persistence of hybrids. Further large-scale studies in other regions and countries are now clearly needed, to better understand these risk factors and the generalisability of our findings to sub-Saharan Africa.^17^, ^19^, ^21^, ^24^, ^27^, ^28^, ^41^, ^45^ Additional work, including modelling studies (to evaluate the role of spillover dynamics in the persistence of *Haematobium* group hybrids) and experimental infections (to compare the infectiousness of different hybrids in *Bulinus* spp) could also have a crucial role in evaluating the potential for hybrids to invade and persist in new geographical locations. Incorporating genomic analysis into future work could provide further insights into the origin, mechanisms, and frequency of novel hybridisations—for example, by improving the crucial distinction between *S haematobium*–*S bovis* and *S haematobium*–*S curassoni* in highly introgressed parasite specimens and optimising identification of F1 hybrids.^19^, ^23^, ^26^, ^27^

Reflecting the current WHO strategies for the control and elimination of schistosomiasis, school-aged children were the focus of our study. As such, sampling of adults was opportunistic, leading to a small sample size and potential biases. Despite this limitation, the fairly large proportion of adults infected with schistosomiasis (and with hybrid schistosomes) in our study remains of interest and importance, as adults are generally dismissed in both research and control programmes. Together with our finding that the zoonotic component of schistosomiasis might be more important than previously assumed, this observation highlights the need for future guidelines to fully consider the contribution of all groups to achieve elimination of schistosomiasis.^13^

In any multi-host parasite system, if elimination is to be achieved, consideration of the contribution of each host species to transmission is essential.^40^, ^46^ In this study we have provided unique insights into the multi-host nature of *S haematobium* group transmission dynamics and the potential implications for control, as well as highlighting the ongoing burden of both human and livestock schistosomiasis in Senegal. Hybridisation of parasites is an emerging public health concern at the interface of infectious disease biology and evolution. Our results demonstrate the complexity and challenges to disease control presented by the multi-host aspect of schistosome transmission in hybrid endemic zones undergoing anthropogenic changes. It will therefore be important to consider evolving host ranges, introgressions, and the wider ecosystem of not only schistosomes, but many other taxonomic groups, and control measures should be considered within a One Health framework if they are to be effective. Against the background of current global trends and our rapidly changing environment, such multi-faceted approaches to the study of infectious diseases will be crucial to inform public health measures locally, nationally, and internationally.

## Data sharing

Molecular sequences from representative samples from the different species were deposited in GenBank (accession numbers MT580946-963, MT579420-449 and MN593376, MN593380, MN593384, MN593388, MN593392, MN593396, MN593400 and MN593404).
