# Supplementary material for: Prevalence and distribution of schistosomiasis in human, livestock, and snail populations in northern Senegal: a One Health epidemiological study of a multi-host system
Source: Lancet Planet Health. 2020 Aug 12;4(8):e330–42. doi: 10.1016/S2542-5196(20)30129-7 (PMC7443702; doi:10.1016/S2542-5196(20)30129-7)
Supplement: Supplementary appendix [file mmc1.pdf]

THE LANCET
Planetary Health

Supplementary appendix

This appendix formed part of the original submission and has been peer reviewed. We post it as supplied by the authors.

Supplement to: Léger E, Borlase A, Fall CB, et al. Prevalence and distribution of schistosomiasis in human, livestock, and snail populations in northern Senegal: a One Health epidemiological study of a multi-host system. *Lancet Planet Health* 2020; **4:** e330–42.

Appendix

S1. Miracidia hatching technique (MHT): human and livestock (adapted from Yu et al. 2007[1]).
Faecal samples: faeces are mixed, weighed (15g for cattle; 5g for small ruminants; 30g or the whole sample for humans) and passed through a 400µm metal sieve into a plastic container (*Schistosoma* eggs pass through this mesh) while rinsing with de-chlorinated water. The filtrate is then passed through both parts of a Pitchford funnel,[2] while agitating and rinsing further. Any eggs present sink to the bottom of the Pitchford funnel and will then be dispensed into a specimen pot with additional de-chlorinated water and placed under light to facilitate sedimentation of any remaining organic material, and hatching of eggs into miracidia. Samples are then examined microscopically for the presence of miracidia.
Tissue samples: tissue samples are first macerated and then processed in the same way as faeces.

S2. DNA extraction and PCR protocols.
Miracidia, cercariae and snail voucher collections were archived with their contextual data in the Schistosomiasis Collection at the Natural History Museum (SCAN).[3]

DNA was extracted from adult worms using the QIAGEN DNeasy Blood & Tissue Kit (QIAGEN, Hilden, Germany), following manufacturer's instructions.
A maximum of 81 (median 11; IQR 22.5) adult worms and eight to 16 miracidia or cercariae per individual host were analysed (or all if <eight available). This strategy was adopted as more robust estimates of genetic diversity within *Schistosoma* populations are found when the number of hosts sampled is increased, rather than the number of samples per host.[4]

DNA amplifications were performed in an Applied Biosystems SimpliAmp™ Thermal Cycler. The PCRs were carried out in a total volume of 25 µl including PuReTaq Ready-To-Go™ PCR Beads (GE Healthcare UK Limited, UK), 0·4 µM of each primer and 3 µL of DNA template.

cox1 primers (5'-3')[5]

Asmit1	TTTTTTGGTCATCCTGAGGTGTAT
SbR	CACAGGATCAGACAAACGAGTACC
ShR	TGATAATCAATGACCCTGCAATAA

PCR conditions: initial denaturing of 5 min at 95°C; 40 cycles of 30 sec at 95°C, 30 sec at 58°C and 1 min at 72°C; final extension period of 7 min at 72°C.

ITS primers (5'-3')[6]

ETTS1	TGCTTAAGTTCAGCGGGT
ETTS2	AACAAGGTTTCCGTAGGTGAA

PCR conditions: initial denaturing of 5 min at 95°C; 40 cycles of 30 sec at 95°C, 30 sec at 58°C and 1 min 30 sec at 72°C; final extension period of 7 min at 72°C.

Four µL of each PCR product were run on 2% GelRed® agarose gel for 45 min at 120 V and visualized on a Synoptics U:Genius GelDoc system.
Selected PCR products were sent to Eurofins Genomics (Cologne, Germany) for purification and sequencing using dilutions of the original PCR primers.

Table S1. Categorisation of genotypes for the *Schistosoma haematobium* group using partial fragment of the mitochondrial cytochrome c oxidase subunit 1 (*cox1* mtDNA) and the complete nuclear ribosomal DNA internal transcribed spacer (ITS rDNA).
Definitive hosts from which miracidia of each category were isolated in this study (and other studies in West Africa) are indicated.

Schistosome species/species combination	Genotype categorisation	*cox1* mtDNA profile	ITS rDNA profile	Definitive host
Schistosoma haematobium	*Sh*	*Sh*	*Sh*	Human
Schistosoma bovis	*Sb*	*Sb*	*Sb*	Cattle, sheep, goat
Schistosoma curassoni	*Sc*	*Sc*	*Sc*	Cattle, sheep, goat
S. haematobium:*S. bovis* hybrid - backcrossed	Hybrid 1 (H1)	*Sb*	*Sh*	Human (rodent[9])
S. haematobium:*S. bovis* hybrid - recent	Hybrid 2 (H2)	*Sb* or *Sh*	Mixed *Sh*:*Sb*	Human
S. bovis:*S. curassoni* hybrid - recent	*Sb*:*Sc*	*Sb* or *Sc*	Mixed *Sb*:*Sc*	Cattle, sheep, goat
S. bovis:*S. curassoni* hybrid - backcrossed	*Sb*:*Sc*	*Sb* or *Sc*	*Sc* or *Sb*	Cattle, sheep, goat (human[19])

S3. Prevalence estimations and estimation of diagnostic test performance in livestock species: Bayesian approaches.

Relatively poor sensitivity of diagnostic tests for human schistosomiasis that rely on egg detection methods are well documented and can lead to biases in estimation of prevalence.[7,8] Bayesian approaches which allow incorporation of uncertainty around diagnostic test performance into adjusted estimation of prevalence have been described for schistosomiasis in human and non-human populations and are advantageous in enabling incorporation, as priors, of additional information regarding model parameters including test sensitivities and specificities.[9,10]

Where one diagnostic test is used, the probability that an individual in a population tests positive for that diagnostic test, *p(Test+)*, is given as:

$$p(Test+) = \pi\, SeTest + (1-\pi)(1-SpTest) \qquad (Model\ 1)$$

Where π is the true prevalence of infection that population, *SeTest* is the test sensitivity and *SpTest* is the test specificity. (Note: This framework was also used for estimating true prevalence in the human and snail surveys).

The Bayesian approach also allows incorporation of results from two or more diagnostic tests such as in the animal survey where the MHT and Kato-Katz (KK) tests have been used in parallel on a randomly selected subset of each animal population. However when diagnostic tests are based on measuring similar biological phenomena, such as is the case for the MHT and KK methods (which are both based on the presence of schistosome eggs in a single faeces sample), the outcomes of the tests for a given individual are likely to be correlated and failing to take this into account may lead to bias in estimates.[11,12]

In this framework the probabilities of an individual testing positive (*Test+*) or negative (*Test-*) for two diagnostic tests (*Test1*, *Test2)*, can be described by the model:

$$p(Test1+, Test2+) = \pi*(SeTest1*SeTest2+covDp) + (1-\pi)*((1-SpTest1)*(1-SpTest2)+covDn)$$
$$p(Test1+, Test2-) = \pi*(SeTest1*(1-SeTest2)-covDp) + (1-\pi)*((1-SpTest1)*SpTest2-covDn)$$
$$p(Test1-, Test2+) = \pi*((1-SeTest1)*SeTest2-covDp) + (1-\pi)*(SpTest1*(1-SpTest2)-covDn)$$
$$p(Test1-, Test2-) = \pi*(1-SeTest1)*(1-SeTest2)+covDp) + (1-\pi)*(SpTest1*SpTest2+covDn)$$
(Model 2)

This enables estimation of schistosomiasis prevalence whilst adjusting for conditional dependence between the two tests, where *covDp* and *covDn* are the covariances between two tests for disease positive and disease negative animals respectively.[11,13]

S4. Estimation of sensitivity for diagnostic tests used in livestock: abattoir data.

Due to lack of data from literature on the performance of diagnostic tests for schistosomiasis in livestock species, abattoir data was used to estimate the sensitivity of KK and MHT as diagnostic tests, and these estimates used to generate priors to be applied to estimation of prevalence using the live animal data.

The sensitivity of a diagnostic test is the proportion of truly positive individuals that are identified as positive by the test. In the absence of a gold-standard diagnostic test for animals for use on abattoir specimens, a pseudo-gold standard was used, whereby any animal positive for worms or miracidia in any tissue or faeces was defined as positive (excluding any animals that did not have a minimum database of results from faeces sample, liver sample and inspection of the mesentery). Sensitivity for each test was then based on the proportion of animals positive on this pseudo-gold standard that were detected by the diagnostic test.

Allowing for the possibility of the post-mortem pseudo-gold standard not detecting all schistosome positive animals, the following adapted form of Model 2 was used to generate all sensitivity estimates for each diagnostic test:

$p(Test+, PM+) = \pi*(SeTest*SePM)+ (1- \pi)*(1-SpTest)*(1-SpPM)$
$p(Test+, PM-) = \pi*(SeTest*(1-SePM)+ (1- \pi)*(1-SpTest)*SpPM$
$p(Test-, PM+) = \pi*(1-SeTest)*SePM + (1- \pi)*SpTest*(1-SpPM)$
$p(Test-, PM-) = \pi *(1-SeTest)*(1-SePM)+ (1- \pi)*(Sp\ Test1*SpTest2)$ *(Model 3)*

With *PM+/-*, *SePM* and *SpPM* representing post-mortem pseudo-gold standard positive/negative and sensitivity/specificity respectively. In this framework π can be considered the probability an animal is truly positive, although it is not used to estimate the prevalence due to biases in the abattoir populations.

Table S2. Bayesian estimate of diagnostic test performance: livestock species.

Median posterior estimate (95% Bayesian credible intervals) for diagnostic test sensitivity: miracidia hatching technique (MHT) and duplicate Kato-Katz slide examination (KK).

**Cattle: *S. bovis*[a]**		**Cattle: *S. curassoni*[b]**		**Goats[c]**		**Sheep[c]**		**Small ruminants (sheep and goats).**	
**MHT**	**KK**	**MHT**	**KK**	**MHT**	**KK**	**MHT**	**KK**	**MHT**	**KK**
**Median posterior (95% BCI)**	**Median posterior (95% BCI)**	**Median posterior (95% BCI)**	**Median posterior (95% BCI)**	**Median posterior (95% BCI)**	**Median posterior (95% BCI)**	**Median posterior (95% BCI)**	**Median posterior (95% BCI)**	**Median posterior (95% BCI)**	**Median posterior (95% BCI)**
0·79 (0·60, 0·91)	0·31 (0·16, 0·49)	0·40 (0·21, 0·62)	0·48 (0·26, 0·70)	0·40 (0·25, 0·57)	0·21 (0·10, 0·36)	0·44 (0·26, 0·64)	0·28 (0·13, 0·47)	0·42 (0·30, 0·54)	0·23 (0·14, 0·34)

[a] Based on animals only found to be infected with *Schistosoma bovis*
[b] Based on animals with *Schistosoma curassoni* infection or *S. curassoni* plus *S. curassoni:S. bovis* hybrid infection.
[c] All schistosome genotype infections.

We identified evidence for a significant difference between the performance of the hatching test in cattle depending on infecting schistosome species, with the proportion of post-mortem positive animals that were MHT positive being higher for those infected with *S. bovis* than those infected with *S. curassoni* +/- *S. bovis*:*S. curassoni* hybrids (Fisher's Exact: p=0·01). Separate sensitivity estimates are therefore presented for cattle. Within sheep and goats, no significant difference in the proportion testing positive was found between those infected with Sc and those infected with *S. bovis* (Fisher's Exact: p=0·56 within sheep; p=0·43 within goats), therefore estimates of test sensitivity for the two small ruminant species are presented which can be considered valid for both genotypes. Furthermore, no significant difference was identified between the proportions of sheep or goats testing positive for each test (infected with *S. bovis* or *S. curassoni*), (Fisher's Exact: p=0·81 MHT; p=0·56 KK), so overall estimates of test performance for small ruminants (sheep and goats) are also presented, which can be considered valid for both species.

S5. Estimation of genotype prevalence.

Given that molecular data was not available for all positive individuals, of those where molecular data were available for each survey (*Nm*), the number testing positive for schistosome genotype i (n_i) was used to estimate the probability (p_i) of a positive individual being infected with that schistosome genotype:

$$n_i \sim dbinom(p_i, Nm) \qquad \textit{(Equation 1)}$$

This was then combined with the adjusted estimation of true prevalence π (using Model 1 or Model 2 depending on dataset) within an adapted Bayesian framework the to give the estimation of prevalence for each genotype (π_i) presented in Figure 2:

$$\pi_i = p_i * \pi \qquad \textit{(Equation 2)}$$

Results plotted in Figure 2 are given in tables S4 and S5.

S6. Priors used in Bayesian frameworks: prevalence estimation.

Prior distributions for test sensitivities and specificities are commonly described using a Beta distribution defined by two coefficients, α and β *(~Beta(α,β)),* as this provides a flexible means of modelling parameters ranging from zero to one. For all estimations of prevalence in the human population, snail population and living livestock populations, uninformative priors were used for the true prevalence *(π~Beta(1,1))*. For the estimation of test sensitivity from the abattoir data, uninformative priors were also used (*SeTest~Beta(1,1)*).

Given that the MHT, KK, cercarial shedding technique and the abattoir pseudo-gold standard are based on microscopic visualisation of clearly identifiable schistosome eggs, miracidia, cercariae or worms, specificity for all tests was set at 100%.

The mean (μ) and standard deviation (σ) of test sensitivity estimates from the abattoir data (Table S1) were used to generate α and β coefficients of the β distribution for use as priors in estimation of the live animal prevalence, where:

$$\alpha = \mu\left(\frac{(1-\mu)\mu}{\sigma^2}\right) - 1 \qquad (\textit{Equation 3})$$

And:

$$\beta = (1-\mu)\left(\frac{(1-\mu)\mu}{\sigma^2} - 1\right) \qquad (\textit{Equation 4})$$

Given the differing profiles of schistosome genotypes found in the live cattle populations of the two sites (Table 3) priors for the diagnostic tests used in the live animal survey estimates of overall prevalence (Table S2) were based on estimates for *S. bovis* for cattle in Richard Toll/Lac de Guiers (RT) and *S. curassoni* for Barkedji (BK) respectively. For sheep and goats, the estimates of test sensitivities for small ruminants was used (Table S2).

Prior distributions for sensitivity of cercarial shedding in snails and of KK and urine filtration methods applied to the human population, were based on literature and expert opinion, with α and β parameters derived using the betaExpert function from the Prevalence package in R based on point estimate and 95% confidence intervals (Table S2). [14]

Following Dendukuri and Joseph (2001),[10] in a Bayesian framework for estimating prevalence using two conditionally dependent tests (such as Model 2 using KK and MHT for livestock), ranges for covariances of disease positive and disease negative animals are specified in the following way:

*0 ≤covDp ≤min(SeTest1,SeTest2)-SeTest1*SeTest2*
*0 ≤covDn ≤min(SpTest1,SpTest2)-SpTest1*SpTest2*

The feasibility range for covariance of disease positive animals was specified in this way as priors in the estimation of livestock prevalence using a uniform distribution. However given that specificity of tests was set at 100%, the framework used did not allow for estimation of the covariance for disease negative animals (*covDn*).

Table S3. Diagnostic test sensitivity estimates and priors for Beta distribution used in estimations of prevalence: duplicate Kato-Katz (KK), miracidia hatching technique (MHT), urine filtration and cercarial shedding.

Species (site)	Test	Sensitivity mean (S.D)	Beta coefficients[a]		Source
			β	α	
**Small ruminants**	KK	0·23 (0·05)	16·06	53·78	Abattoir data
	MHT	0·42 (0·06)	28	38·67	
**Cattle (Richard Toll)**	KK	0·31 (0·08)	10·05	22·37	
	MHT	0·78 (0·08)	20·13	5·68	
**Cattle (Barkedji)**	KK	0·40 (0·106)	8.23	12·18	
	MHT	0·478 (0·112)	9·01	9·88	

Species	Test	Sensitivity estimate (range)	Beta coefficients[b]		Source
			β	α	
**Humans**	Urine filtration	0·81 (0·78-0·85)	414·48	97·99	King and Bertsch 2013[15]
**Humans**	KK	0·75 (0·5-0·99)	12·83	4·94	Lamberton et al. 2014[16]; Barenbold et al. 2017, 2018[17,18]; Koukounari et al. 2013[19]
**Snails**	Cercarial shedding	0·95 (0·9-1)	120·19	7·27	Authors personal observation.

[a]Derived by fitting Beta distribution to mean and standard deviation (S.D).
[b]Derived using BetaExpert package, point estimate and range.

Table S4. Bayesian estimation of prevalence by schistosome genotype: human surveys (as plotted in Figure 2 main text).

Survey	Age group	Schistosome Species	Median posterior prevalence (Range; IQR)
**Richard Toll/ LDG 2016**	Children	*S. haematobium*	0.81 (0.74-0.88;0.78-0.83)
		S. haematobium-S. bovis hybrids (H1 & H2)	0.55 (0.47-0.62; 0.52-0.57)
	Adults	*S.haematobium*	0.65 (0.34-0.91; 0.55-0.76)
		S. haematobium-S. bovis hybrids (H1 & H2)	0.38 (0.11-0.71; 0.27-0.5)
**Richard Toll/ LDG 2017-18**	Children	*S. haematobium*	0.8 (0.73-0.87; 0.77-0.82)
		S. haematobium-S. bovis hybrids (H1 & H2)	0.63 (0.56-0.71; 0.61-0.66)
	Adults	*S.haematobium*	0.33 (0.21-0.46; 0.29-0.38)
		S. haematobium-S. bovis hybrids (H1 & H2)	0.38 (0.27-0.52; 0.34-0.4)
**Barkedji 2016**	Children	*S. haematobium*	0.29 (0.22-0.37; 0.26-0.32)
		S. haematobium-S. bovis hybrids (H1 & H2)	0.04 0.01-0.08; 0.03-0.05)
	Adults	*S.haematobium*	0.24 0.16-0.34; 0.21-0.28)
		S. haematobium-S. bovis hybrids (H1 & H2)	0.06 (0.02-0.13; 0.04-0.08)
**Barkedji 2017-18**	Children	*S. haematobium*	0.41 (0.34-0.48; 0.39-0.43)
		S. haematobium-S. bovis hybrids (H1 & H2)	0.04 (0.02-0.08; 0.03-0.06)
	Adults	*S.haematobium*	0.44 (0.31-0.58;0.4-0.49)
		S. haematobium-S. bovis hybrids (H1 & H2)	0.02 (0-0.09; 0.01-0.04)

Table S5. Bayesian estimation of prevalence by schistosome genotype: livestock surveys (as plotted in Figure 2 main text).

Survey	Livestock Species	Schistosome Species	Median posterior prevalence (Range; IQR)
**Richard Toll/ LDG 2017**	Cattle	*S. bovis*	0.92 (0.8-0.99; 0.89-0.96)
		S. curassoni	0.01 (0-0.04; 0-0.01)
		S. bovis- S. curassoni hybrids	0.02 (0-0.06; 0.01-0.03)
	Goats	*S. bovis*	0.15 (0.05-0.33; 0.1-0.2)
		S. curassoni	0.11 (0.03-0.27; 0.07-0.15)
		S. bovis- S. curassoni hybrids	0.03 (0-0.14; 0.01-0.06)
	Sheep	*S. bovis*	0.14 (0.05-0.31; 0.11-0.19)
		S. curassoni	0.01 (0-0.07; 0.01-0.03)
		S. bovis- S. curassoni hybrids	0.01 (0-0.08; 0.01-0.03)
**Barkedji 2017**	Cattle	*S. bovis*	0.02 (0-0.12; 0.01-0.04)
		S. curassoni	0.08 (0.02-0.24; 0.05-0.11)
		S. bovis- S. curassoni hybrids	0.02 (0-0.12; 0.01-0.04)
	Goats	*S. bovis*	0.02 (0-0.09; 0.01-0.04)
		S. curassoni	0.84 (0.61-0.98; 0.76-0.91)
		S. bovis- S. curassoni hybrids	0.02 (0-0.09; 0.01-0.04)
	Sheep	*S. bovis*	0.02 (0-0.08; 0.01-0.03)
		S. curassoni	0.73 (0.48-0.93; 0.64-0.81)
		S. bovis- S. curassoni hybrids	0.04 (0.01-0.12; 0.02-0.06)

References

1. Yu JM, de Vlas SJ, Jiang QW, Gryseels B. Comparison of the Kato-Katz technique, hatching test and indirect hemagglutination assay (IHA) for the diagnosis of *Schistosoma japonicum* infection in China. *Parasitology International* 2007; **56**(1): 45-9.
2. Pitchford RJ, Visser PS. A simple and rapid technique for quantitative estimation of helminth eggs in human and animal excreta with special reference to *Schistosoma sp*. *Transactions of The Royal Society of Tropical Medicine and Hygiene* 1975; **69**(3): 318-22.
3. Emery A, Allan F, Rabone M, Rollinson D. Schistosomiasis Collection at NHM (SCAN). *Parasites & Vectors* 2012; 5:185.
4. French MD, Churcher TS, Basanez MG, Norton AJ, Lwambo NJS, Webster JP. Reductions in genetic diversity of *Schistosoma mansoni* populations under chemotherapeutic pressure: the effect of sampling approach and parasite population definition. *Acta Tropica* 2013; 128(2): 196-205.
5. Webster BL, Rollinson D, Stothard JR, Huyse T. Rapid diagnostic multiplex PCR (RD-PCR) to discriminate *Schistosoma haematobium* and *S. bovis*. *Journal of Helminthology* 2010; **84**(1): 107-14.
6. Kane RA, Rollinson D. Repetitive sequences in the ribosomal dna internal transcribed spacer of *Schistosoma-haematobium*, *Schistosoma-intercalatum* and *Schistosoma-mattheei*. *Molecular and Biochemical Parasitology* 1994; **63**(1): 153-6.
7. Kongs A, Marks G, Verle P, Van Der Stuyft P. The unreliability of the Kato-Katz technique limits its usefulness for evaluating *S. mansoni* infections. *Tropical Medicine & International Health* 2001; **6**(3): 163-9.

8. Savioli L, Hatz C, Dixon H, Kisumku UM, Mott KE. Control of morbidity due to *Schistosoma haematobium* on Pemba Island: egg excretion and hematuria as indicators of infection. *American Journal of Tropical Medicine and Hygiene* 1990; **43**(3): 289-95.
9. Rudge JW, Webster JP, Lu DB, Wang TP, Fang GR, Basanez MG. Identifying host species driving transmission of schistosomiasis japonica, a multihost parasite system, in China. *Proceedings of the National Academy of Sciences of the United States of America* 2013; **110**(28): 11457-62.
10. Wang X-H, Wu X-H, Zhou X-N. Bayesian estimation of community prevalences of *Schistosoma japonicum* infection in China. *International Journal for Parasitology* 2006; **36**(8): 895-902.
11. Dendukuri N, Joseph L. Bayesian approaches to modeling the conditional dependence between multiple diagnostic tests. *Biometrics* 2001; **57**(1): 158-67.
12. Gardner IA, Stryhn H, Lind P, Collins MT. Conditional dependence between tests affects the diagnosis and surveillance of animal diseases. *Preventive Veterinary Medicine* 2000; **45**(1-2): 107-22.
13. Branscum A, Gardner I, Johnson W. Estimation of diagnostic-test sensitivity and specificity through Bayesian modeling. *Preventive Veterinary Medicine* 2005; **68**(2-4): 145-63.
14. Devleesschauwer B, Torgerson P, Charlier J, et al. Prevalence : Tools for assessing prevalence assessment studies. 2014. R package version 0.4.0. http://cran.r-project.org/package=prevalence
15. King CH, Bertsch D. Meta-analysis of urine heme dipstick diagnosis of *Schistosoma haematobium* infection, including low-prevalence and previously-treated populations. *PLoS Neglected Tropical Diseases* 2013; **7**(9): e2431.
16. Lamberton PH, Kabatereine NB, Oguttu DW, Fenwick A, Webster JP. Sensitivity and specificity of multiple Kato-Katz thick smears and a circulating cathodic antigen test for *Schistosoma mansoni* diagnosis pre- and post-repeated-praziquantel treatment. *PLoS Neglected Tropical Diseases* 2014; **8**(9): e3139.
17. Bärenbold O, Raso G, Coulibaly JT, N'Goran EK, Utzinger J, Vounatsou P. Estimating sensitivity of the Kato-Katz technique for the diagnosis of *Schistosoma mansoni* and hookworm in relation to infection intensity. *PLoS Neglected Tropical Diseases* 2017; **11**(10): e0005953.
18. Bärenbold O, Garba A, Colley DG, et al. Translating preventive chemotherapy prevalence thresholds for Schistosoma mansoni from the Kato-Katz technique into the point-of-care circulating cathodic antigen diagnostic test. *PLoS Neglected Tropical Diseases* 2018; **12**(12): e0006941.
19. Koukounari A, Donnelly CA, Moustaki I, et al. A latent Markov modelling approach to the evaluation of circulating cathodic antigen strips for schistosomiasis diagnosis pre-and post-praziquantel treatment in Uganda. *PLoS computational biology* 2013; **9**(12): e1003402.
